# Risk factors for airway clearance dysfunction in children with severe pneumonia: a retrospective study of LASSO model

**DOI:** 10.3389/fped.2025.1638103

**Published:** 2025-10-29

**Authors:** Xiaoyan Ding, Jing Bai, Rui Liu

**Affiliations:** Department of Pediatrics, Beijing Anzhen Nanchong Hospital of Capital Medical University & Nanchong Central Hospital, Nanchong, Sichuan, China

**Keywords:** children with severe pneumonia, airway clearance dysfunction, mechanical ventilation, c-Reactive protein, ineffective cough

## Abstract

**Background:**

This study aimed to investigate the types and risk factors of airway clearance dysfunction (ACD) in children with severe pneumonia based on LASSO regression and multivariate *Logistic* regression analysis.

**Methods:**

This study was approved by the Hospital Ethics Committee. A retrospective study was conducted on 147 children with severe pneumonia admitted between January 2024 and October 2024. Demographic and clinical characteristics were collected, and the incidence and types of ACD were analyzed. Patients with ACD were assigned to the ACD group, while those without ACD were assigned to the non-ACD group. LASSO regression and multivariate *Logistic* regression were used to identify risk factors for ACD in these children, and ROC curves were constructed to evaluate the predictive value of these factors for ACD.

**Results:**

A total of 63 cases of ACD were observed among children with severe pneumonia, including ineffective coughing, thick sputum, dyspnea, nasal flaring/ increased nasal congestion, tachypnea, and oxygen saturation <93%, with an incidence rate of 42.86%. Significant differences were found between the ACD group and non-ACD group in terms of age, mechanical ventilation, nutritional status, comorbid respiratory failure, comorbid heart failure, pulmonary rales, and pulmonary rhonchi (*P* < 0.05). Additionally, the ACD group had lower platelet count (PLT), immunoglobulin A (IgA), and immunoglobulin M (IgM) levels, while C-reactive protein (CRP), procalcitonin (PCT), and interleukin-6 (IL-6) levels were higher compared to the non-ACD group (*P* < 0.05). Variable screening was performed using the LASSO regression model, identifying three significant influencing factors. These were incorporated into a *Logistic* regression model, which revealed that mechanical ventilation, CRP, PCT, IL-6, and IgA were influencing factors for ACD in children with severe pneumonia. Based on these findings, an ROC curve was constructed, demonstrating that the combined prediction of mechanical ventilation, CRP, PCT, IL-6, and IgA for ACD in children with severe pneumonia achieved an AUC of 0.882, significantly higher than the AUC of any single indicator (*P* < 0.05).

**Conclusion:**

Children with severe pneumonia are at risk of developing ACD, which may be influenced by mechanical ventilation, CRP, PCT, IL-6, and IgA levels. These five factors can be used to assess the risk of ACD in children with severe pneumonia. Accordingly, clinical measures should be developed to improve airway clearance ability and promote recovery in these patients.

## Introduction

Severe pneumonia is a common critical emergency in pediatrics. Due to children's underdeveloped immune systems, poor cardiopulmonary function, and generally weaker physical condition, the morbidity and mortality rates of severe pneumonia are higher in children compared to other populations (1), posing a significant threat to their lives. According to World Health Organization statistics (2), severe pneumonia accounts for approximately 7%–13% of all pediatric pneumonia cases and is a major cause of death in children. Studies have shown (3) that patients with severe pneumonia often experience airway clearance dysfunction (ACD), which can exacerbate respiratory dysfunction and even lead to serious complications such as atelectasis and worsened infections, hindering rapid recovery. ACD refers to the inability to clear respiratory secretions or obstructions to maintain airway patency. Without timely and targeted intervention, it may result in hypoxia, respiratory failure, aggravated local ischemia in the heart and brain, and even immediate death (4). Relevant studies have found (5) that airway clearance is closely associated with the mucociliary clearance system and an effective cough mechanism. Cilia facilitate the transport of secretions from central to periphery airways through beating motions, while coughing expels secretions via high- velocity airflow. However, children with severe pneumonia often exhibit intense inflammatory responses that can alter mucus properties, leading to thickened secretions that are difficult to expel. This significantly impairs ciliary clearance and may cause weak or absent cough reflexes, ultimately resulting in secretion retention and an increased incidence of ACD (6, 7).

Currently, there are various clinically observed types of ACD in children with severe pneumonia, among which ineffective cough, thick sputum, and dyspnea are commonly seen, significantly impacting treatment and recovery. Ineffective cough, a major manifestation of ACD in these patients, serves as a clinical predictor of impaired airway clearance. It is often associated with weak cough reflexes or airway narrowing, preventing effective expulsion of secretions. Thick sputum is another contributing factor to ACD; its presence increases the risk of inadequate airway clearance and may even worsen airway secretions, further compromising respiratory function. Dyspnea, also a critical issue in ACD among severe pneumonia patients, may result from multiple factors such as retained airway secretions, airway blockage, and pulmonary infection. Additionally, excessive phlegm or increased mucus secretion in these patients can significantly exacerbate breathing difficulties (8, 9). If not effectively managed during hospitalization, ACD can lead to alveolar damage, atelectasis, pulmonary edema, and in severe cases, may progress to acute respiratory distress syndrome (ARDS) or multiple organ failure. These complications directly delay recovery and may even increase in-hospital mortality (10, 11).

In light of this, the present study was conducted to early identify the types and risk factors of ACD in children with severe pneumonia. The objective is to elucidate the categories and underlying risk factors of ACD in pediatric severe pneumonia, thereby providing a scientific basis for developing more effective treatment strategies.

## Methods

### Research objects

This study has been reviewed by the ethical committee of the hospital. Using a retrospective research approach, 147 children with severe pneumonia were admitted between January 2024 and October 2024. Demographic and clinical characteristics were collected, and the incidence and type of ACD were analyzed. Patients who developed ACD were assigned to the ACD group, while those without ACD were assigned to the non-ACD group.

### Inclusion and exclusion criteria

Inclusion criteria: (1) compliance with WHO guidelines for the management of severe pneumonia in children (12); (2) diagnosis confirmed by chest x-ray, CT, and other imaging examinations; (3) age <13 years; (4) complete medical record data and follow-up information; (5) normal cognition and no communication barriers.

Exclusion Criteria: (1) unstable vital signs or death during hospitalization; (2) basic diseases of blood system or immunodeficiency-based diseases; (3) presence of congenital diseases; (4) transferring to another hospital during the study or withdrawing on their own for any reason.

### Patient demographics

Demographic and clinical characteristics of children with severe pneumonia were collected based on the medical record information management system, including age, gender, body mass index (BMI), body temperature, time from symptom onset to hospital visit, length of hospital stay, place of residence, congenital disease, mechanical ventilation, nutritional status, comorbid respiratory failure, comorbid heart failure, pulmonary rales, pulmonary rhonchi, use of sedative/analgesic drugs, and oxygen therapy modalities.

### Laboratory indicators

On the day of testing, peripheral venous blood specimens (3 ml) were collected from the children and kept indoors for 15 minutes, then centrifuged at 3,000 r/min for 10 minutes. The supernatant serum was collected for subsequent analyses. White blood cell count (WBC) and platelet count (PLT) were measured using a D5-CRP fully automated hematology analyzer (Shenzhen Dimension Bio-Technology Co., Ltd.) with its matching reagents. C-reactive protein (CRP), procalcitonin (PCT), and interleukin-6 (IL-6) levels were determined by enzyme-linked immunosorbent assay (ELISA). Additionally, hemoglobin (Hb) and albumin (Alb) were quantified colorimetrically using an AU5800 fully automated biochemical analyzer (Beckman, USA).

### Immunological indicators

On the day of testing, peripheral venous blood samples (3 ml) of children were collected. The samples were allowed to stand at room temperature for 15 minutes, followed by centrifugation at 3,000 r/min for 10 minutes to separate the serum. Levels of immunoglobulin G (IgG), immunoglobulin A (IgA), and immunoglobulin M (IgM) were measured in strict accordance with the immunoturbidimetric method and instructions of the assay kits (Tianjin Fremid Biomedical Technology Co., Ltd.).

### Statistical analysis

Data analysis in this study was performed using SPSS 25.0 statistical software. Categorical data are presented as *n* (%), and were analyzed using the *χ*^2^ test. Measurement data were tested for normality with the Shapiro–Wilk test. Normal distributed data are expressed as mean ± standard deviation(SD), with comparison between two groups performed by independent samples *t-*test, and comparison between the two groups were conducted using independent samples *t*-test. Non- normally distributed data are expressed as [M (P_25_, P_75_)] and analyzed using the nonparametric Mann–Whitney *U*-test. LASSO regression and multivariate logistic regression were employed to analyze risk factors for ACD in children with severe pneumonia. Receiver operating characteristic (ROC) curves were constructed to evaluate the predictive value of these factors for ACD. A *P* < 0.05 was considered statistically significant.

## Results

### Types of ACD in children with severe pneumonia

All 147 children with severe pneumonia received broad-spectrum antibiotics for anti-infection treatment, along with antiviral therapy, anti-shock management, cough relief and bronchodilation, oxygen inhalation, and nutritional support after admission. The incidence and types of ACD were evaluated, including 9 cases of ineffective cough, 18 cases of thick sputum, 6 cases of dyspnea, 11 cases of nasal flaring/increased congestion, 7 cases of tachypnea, and 12 cases of oxygen saturation <93%. A total of 63 children were assigned to the ACD group, corresponding to an ACD incidence of 42.86%. The remaining 84 children without ACD were assigned to the non-ACD group (Table 1).

**Table 1:** Types of ACD in children with severe pneumonia (*n* = 147).

ACD types	Cases/*n*	Percentage/%
Ineffective cough	9	6.12
Thick sputum	18	12.24
Dyspnea	6	4.08
Nasal flaring/increased nasal congestion	11	7.48
Tachypnea	7	4.76
Oxygen saturation <93%	12	8.16
Ttotal	63	42.86

### Demographic and clinical characteristics of children in the ACD and non-ACD groups

To compare the demographic and clinical characteristics between the two groups, the results showed that the mean age of the ACD group was 4.87 years, significantly younger than that of the non-ACD group (5.49 years; *P* < 0.05). The proportion of patients receiving mechanical ventilation in the ACD group was 68.25%, which was significantly higher than that in the non-ACD group (30.95%; *P* < 0.05). The prevalence of malnutrition in the ACD group was 73.02%, markedly higher than that in the non-ACD group (30.95%; *P* < 0.05). There were 41 cases of comorbid respiratory failure in the ACD group, compared to only 20 cases in the non-ACD group, indicating a significantly higher incidence in the ACD group (*P* < 0.05). Similarly, the ACD group had 45 cases of comorbid heart failure, significantly more than the 28 cases in the non-ACD group (*P* < 0.05). Pulmonary rales were observed in 61.90% of the ACD group, a higher proportion than in the non-ACD group (41.67%; *P* < 0.05). The incidence of pulmonary rhonchi in the ACD group was 69.84%, significantly higher than that in the non-ACD group (35.71%; *P* < 0.05). However, no significant differences were observed in BMI (ACD group: 22.75 vs. non-ACD group: 23.05; *P* > 0.05), mean body temperature (ACD group: 38.91°C vs. non-ACD group: 38.74°C; *P* > 0.05), time from symptom onset to hospital visit (ACD group: 5.27 days vs. non-ACD group: 5.09 days; *P* > 0.05), or length of hospital stay (ACD group: 11.41 days vs. non-ACD group: 10.97 days; *P* > 0.05). Additionally, no significant differences were found between the two groups in terms of gender, place of residence, congenital diseases, use of sedative/analgesic drugs, or oxygen therapy modalities (*P* > 0.05, Table 2).

**Table 2:** Demographic data and clinical characteristics of children in the ACD and non-ACD groups (*n* = 147).

Clinical data		ACD group (*n* = 63)	Non-ACD group (*n* = 84)	*χ^2^*/*t*	*P*
Age (years)		4.87 ± 1.42	5.49 ± 1.81	2.248	0.026
Gender	Male	36	45	0.186	0.667
	Female	27	39		
BMI (kg/m^2^)		22.75 ± 1.62	23.05 ± 1.59	1.123	0.263
Body temperature (°C)		38.91 ± 0.62	38.74 ± 0.58	1.707	0.090
Time from onset to hospital visit (d)		5.27 ± 1.45	5.08 ± 1.23	0.858	0.392
Length of hospital stay (d)		11.41 ± 3.38	10.96 ± 3.15	0.831	0.408
Place of residence	Countryside	22	31	0.061	0.804
	Town	41	53		
Congenital disease	Yes	9	13	0.040	0.841
	No	54	71		
Mechanical ventilation	Yes	43	26	20.112	<0.001
	No	20	58		
Nutritional status	Normal	17	58	25.489	<0.001
	Dystrophy	46	26		
Comorbid respiratory failure	Yes	41	20	25.257	<0.001
	No	22	64		
Comorbid heart failure	Yes	45	28	20.899	<0.001
	No	18	56		
Pulmonary rales	Yes	39	35	5.898	0.015
	No	24	49		
Pulmonary rhonchi	Yes	44	30	16.772	<0.001
	No	19	54		
Use of sedative/analgesic drugs	Yes	15	18	0.117	0.732
	No	48	66		
Oxygen therapy	Head mask oxygenation	27	35	0.021	0.885
	Noninvasive assisted ventilation	36	49		

### Comparison of laboratory indicators between the ACD and non-ACD groups

To compare the laboratory indicators between the two groups, the results showed that the PLT level in the ACD group was 182.45, significantly lower than that in the non-ACD group (203.17; *P* < 0.05). The CRP level in the ACD group was 34.02, markedly higher than that in the non-ACD group (27.26; *P* < 0.05). The PCT level in the ACD group was 4.08, higher than that in the non-ACD group (3.36; *P* < 0.05). The IL-6 level in the ACD group was 15.27, significantly elevated compared to the non-ACD group (13.19; *P* < 0.05).

However, no significant differences were observed in WBC (ACD group: 8.71 vs. non-ACD group: 8.59; *P* > 0.05), Hb level (ACD group: 105.64 vs. non-ACD group: 106.19; *P* > 0.05), or Alb level (ACD group: 35.29 vs. non-ACD group: 36.34; *P* > 0.05, Table 3).

**Table 3:** Laboratory indicators of children in the ACD and non-ACD groups (*n* = 147).

Laboratory indicators	ACD group (*n* = 63)	Non-ACD group (*n* = 84)	*χ^2^*/*t*	*P*
WBC (×10^9^/L)	8.71 ± 2.89	8.59 ± 2.81	0.253	0.801
PLT (×10^9^/L)	182.45 ± 20.36	203.17 ± 22.03	5.828	<0.001
CRP (mg/L)	34.02 ± 8.56	27.26 ± 7.03	5.253	<0.001
Hb (g/L)	105.64 ± 12.31	106.19 ± 12.82	0.262	0.794
Alb (g/L)	35.29 ± 4.59	36.34 ± 4.81	1.336	0.184
PCT (µg/L)	4.08 ± 1.35	3.36 ± 1.11	3.546	0.001
IL-6 (ng/L)	15.27 ± 5.06	13.19 ± 4.05	2.767	0.006

### Comparison of immunological indicators between the ACD and non-ACD groups

**Figure 1:**
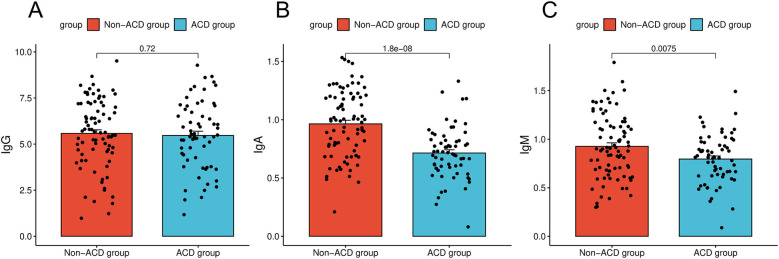
Comparison of immunologic indices between children in the ACD and non-ACD groups. **(A)** Comparison of immunologic indices between children in the ACD and IgG. **(B)** Comparison of immunologic indices between children in the ACD and IgA. **(C)** Comparison of immunologic indices between children in the ACD and IgM.

The immunological indicators were compared between the two groups. The results showed that the IgA level in the ACD group was 0.71, significantly lower than that in the non-ACD group (0.96; *P* < 0.05). The IgM level in the ACD group was 0.80, which was also lower than that in the non-ACD group (0.93; *P* < 0.05). However, no significant difference was observed in immunoglobulin G (IgG) levels between the ACD group (5.47) and the non-ACD group (5.58; *P* > 0.05, Figure 1).

### Screening of univariate factors influencing ACD using LASSO regression

**Figure 2:**
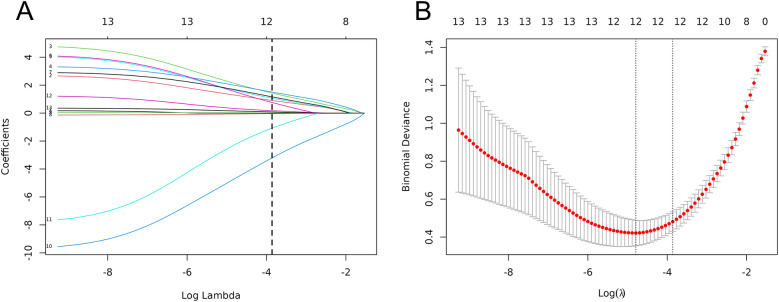
Clinical feature selection based on LASSO regression models. **(A)** LASSO coeffcient profles. **(B)** Three risk factors were selected using LASSO Cox regression analysis.

With the occurrence of ACD in children with severe pneumonia as the dependent variable, the LASSO regression model was employed to screen potential influencing factors. Variables were selected from 13 candidate factors (age, mechanical ventilation, nutritional status, comorbid respiratory failure, comorbid heart failure, pulmonary rales, pulmonary rhonchi, PLT, CRP, PCT, IL-6, IgA, and IgM). The Ten-fold cross-validation was used to identify the optimal λ value. As the penalty coefficients λ varied, the coefficients of the independent variables were gradually compressed (Figure 2A). The λ value corresponding to the minimum cross-validation error (λ = 0.021) was ultimately selected as the optimal value (details in Figure 2B). This process identified five significant influencing factors: mechanical ventilation, CRP, PCT, IL-6, and IgA.

### Construction of a predictive model for ACD in children with severe pneumonia using LASSO regression

The five factors selected by the LASSO regression model were incorporated into a *Logistic* regression model. Stepwise regression was then applied to identify influencing factors for ACD in children with severe pneumonia. The results demonstrated that mechanical ventilation (OR = 4.80), CRP (OR = 1.12), PCT (OR = 1.65), IL-6 (OR = 1.11), and IgA (OR = 0.02) were the independent influencing factors for ACD in children with severe pneumonia (Table 4).

**Table 4:** Logistic regression analysis of ACD occurrence in children with severe pneumoni**a.**

Factors	Regression coefficients	Standard error	*Z* value	*P* value	OR value	OR value 95% CI
Mechanical ventilation	1.57	0.36	4.37	<0.001	4.80	2.37–9.70
CRP	0.11	0.03	4.48	<0.001	1.12	1.07–1.18
PCT	0.50	0.15	3.29	0.001	1.65	1.22–2.21
IL-6	0.10	0.04	2.64	0.008	1.11	1.03–1.19
IgA	-3.78	0.79	−4.77	<0.001	0.02	0.00–0.11

### ROC curve analysis

**Figure 3:**
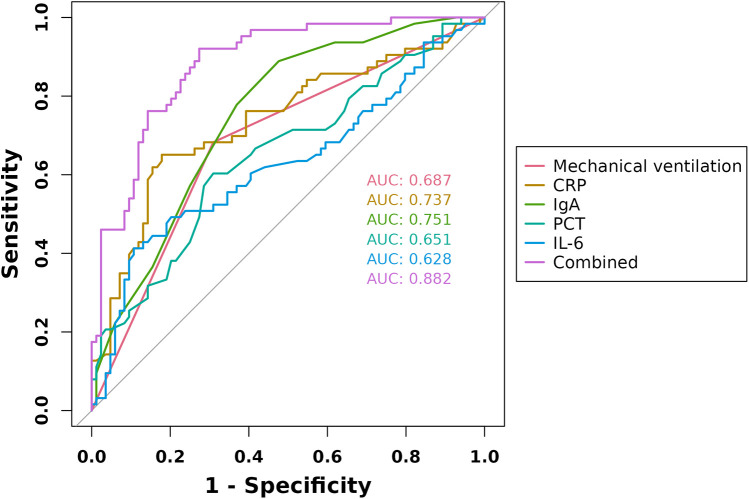
ROC curves of mechanical ventilation, CRP, PCT, IL-6, and IgA in predicting ACD in children with severe pneumonia.

ROC curves were constructed for the five factors—mechanical ventilation, CRP, PCT, IL-6, and IgA—to evaluate their predictive value for ACD in children with severe pneumonia. The results showed that the AUC values for predicting ACD using mechanical ventilation, CRP, PCT, IL-6, and IgA were 0.687, 0.737, 0.651, 0.628, and 0.751, respectively. In contrast, the combined predictive AUC reached 0.882, which was significantly higher than that of any single indicator (*P* < 0.05), indicating that the combination of these factors has high predictive value for ACD in children with severe pneumonia (Figure 3).

## Discussion

Studies have indicated (13) that 40%–60% of children with severe pneumonia are prone to producing large amounts of thick sputum, which not only increases the risk of airway obstruction but also elevates the likelihood of ACD. Severe pneumonia in children often involves respiratory system compromise, with clinical manifestations including cough, tachypnea, increased respiratory secretions, and may also be accompanied by severe dyspnea, nasal flaring, and cyanosis (14, 15). Additionally, due to their generally weaker physical condition, respiratory infections can lead to excessive secretions, making it difficult for children to effectively expectorate sputum on their own, thereby increasing the incidence of ACD and posing significant challenges in clinical management.

In this study, the types of ACD observed in children with severe pneumonia primarily included ineffective cough, thick sputum, dyspnea, nasal flaring/increased nasal congestion, tachypnea, and oxygen saturation <93%., Among these, thick sputum was the most common type, and the overall incidence of ACD reached 42.86%, which is consistent with clinical data. Severe pneumonia is often accompanied by varying degrees of inflammatory response, which can directly stimulate goblet cells and submucosal glands to overproduce mucin and alter mucus composition, thereby increasing its viscoelasticity and viscosity. Meanwhile, inflammatory mediators can damage airway ciliated epithelium, reducing the frequency and efficiency of coordinated ciliary beating. Moreover, young patients have underdeveloped organ function, which often leads to weak or ineffective coughing and an inability to generate sufficient airflow shear force to clear secretions, further contributing to the high incidence of ACD. Therefore, early recognition of ACD and implementation of targeted interventions are crucial.

In this study, it was observed that the children in the ACD group were younger than those in the non-ACD group, suggesting that younger children are more susceptible to ACD. This is primarily due to their immature respiratory system, relatively narrow airways, poor ciliary motility, and underdeveloped cough reflex, all of which increase the risk of ACD. Additionally, the nutritional status of ACD group was poorer than that of the non-ACD group. Malnutrition can compromise immune function, weaken respiratory muscle strength, impair mucosal repair capacity, adversely affect mucus composition and quality, and even lead to deteriorated pulmonary function and impaired respiratory muscle contraction. These factors can exacerbate infection and thereby increase the risk of ACD manifestations such as ineffective cough and thick sputum. The incidence of comorbid respiratory failure, heart failure, pulmonary rales, and pulmonary rhonchi was significantly higher in the ACD group than in the non-ACD group. This indicates that children with severe pneumonia who present with these complications are at a markedly elevated risk of developing ACD. Respiratory failure significantly impairs lung function, directly hinders gas exchange, and reduces cough efficacy, thereby compromising airway clearance. Heart failure can cause pulmonary congestion and edema, increasing the likelihood of lung pathology and ACD. Pulmonary rales and rhonchi are both signs of accumulated airway secretions, suggesting either excessive airway secretions or airway narrowing, which further raises the risk of impaired airway clearance.

Studies have shown that mechanical ventilation, an invasive procedure used to support respiratory function through an artificial airway, can disrupt the natural defense mechanisms of the respiratory tract. It impedes ciliary movement, reduces mucus clearance, and promotes the accumulation of airway secretions, thereby worsening ACD (16, 17). Consistent with this, the current study found a significantly higher rate of mechanical ventilation in the ACD group, confirming it as a risk factor for ACD in children with severe pneumonia. Analysis indicates that mechanical ventilation requires endotracheal intubation or tracheotomy, reflecting the critical condition of the children and pronounced respiratory symptoms. Prolonged mechanical ventilation can impair spontaneous breathing, promote excessive secretions retention, weaken the cough reflex, and diminish the autonomous cleaning ability of the airways, ultimately increasing the incidence of ACD (18, 19). In light of these findings, clinical practice should emphasize enhanced airway management for mechanically ventilated children with severe pneumonia, including timely clearance of respiratory secretions, to maintain airway patency.

Typically, children with severe pneumonia release large amounts of inflammatory mediators and cytokines, making them highly significant in assessing the severity of the disease (20). Changes in PLT often reflect the intensity of the inflammatory response. An elevated PLT level indicates a serious inflammatory reaction or abnormal coagulation, which is commonly observed in children with severe pneumonia. CRP, a nonspecific acute-phase inflammatory marker, is associated with the degree of systemic inflammation and tissue damage. Its elevated expression in children with severe pneumonia suggests active inflammation and a higher risk of pulmonary complications. PCT, a prohormone of calcitonin without hormonal activity, increases significantly in response to severe stimuli such as bacterial infection, making it a key indicator for clinical identification of bacterial infections. IL-6, secreted by various cells including T lymphocytes, fibroblasts, B lymphocytes, and monocytes/macrophages, plays a critical role in inflammatory and immune responses, serving as an important marker for early diagnosis and warning of infectious diseases (21). In this study, the ACD group exhibited lower PLT levels but significantly higher CRP, PCT, and IL-6 levels compared to the non-ACD group. This suggests that PLT and CRP expression may influence the occurrence of ACD, with CRP, PCT, and IL-6 being particularly significant factors contributing to ACD in children with severe pneumonia. Although PLT was significantly lower in the ACD group, it was not selected in the final LASSO model, possibly due to its complex relationship with inflammation and coagulation, which may have been overshadowed by more direct inflammatory markers such as CRP and PCT, or due to multicollinearity. Elevated CRP levels are closely related to airway mucosal damage and impaired mucociliary clearance. As CRP increases, severe damage to the airway mucosa, dysfunction of ciliary clearance, and epithelial cell shedding may occur, leading to the formation of mucus plugs that obstruct the airways. Additionally, elevated CRP can contribute to the formation of plastic mucus, further increasing the risk of airway obstruction and ACD. Similarly, elevated PCT levels in children with severe pneumonia often indicate severe infection, which may be accompanied by intense inflammatory responses and tissue damage, resulting in increased mucus secretion, impaired mucociliary function, and ultimately ACD. As a pro-inflammatory cytokine, IL-6 is often elevated in severe pneumonia, reflecting exacerbated airway inflammation, increased mucus production, and impaired ciliary function, all of which significantly raise the incidence of ACD (22, 23). Therefore, close clinical monitoring of CRP, PCT, and IL-6 levels is essential. Regular assessment of these markers can help identify the risk of ACD in children with severe pneumonia at an early stage, enabling timely intervention.

It has been found (24) that children with severe pneumonia are often associated with immune dysfunction, while the immune function of children directly affects the severity of this disease. Immune dysfunction is manifested as abnormalities in cellular and humoral immunity, and several studies have shown that compared with children with simple pneumonia, children with severe pneumonia have relatively lower levels of IgA and IgM, with their condition worsening as the levels of indicators decrease (25). Among them, IgM is the earliest type of antibody produced in the immune response, which activates the complement system and enhances phagocytosis of pathogens, and in children with severe pneumonia, the changes of its level mainly reflect the acute phase characteristics of pathogen infection; while IgA, as a key factor of mucosal local immunity, tends to be distributed on the mucosal surfaces of respiratory and digestive tracts, which can play the role of anti-infection and protective barrier. The reduced IgA expression in children with severe pneumonia indicates that their respiratory mucosal defense ability is attenuated, and children are highly susceptible to the attack of pathogens (26, 27). From the results of this study, the levels of IgA and IgM were significantly lower in ACD group than in non-ACD group, and especially IgA as a key factor in the development of ACD in children with severe pneumonia. It has been reported (28) that IgA is the main protective antibodies of respiratory mucosa, which can directly inhibit the adhesion of pathogens on respiratory epithelial cells, thus avoiding pathogens from colonizing and infecting. When the level of its expression decreases, the ability of body to resist the pathogens is clearly weakened and even results in recurrent infections and intensification of inflammatory reactions, which in turn causes ACD. In addition, reduced IgA levels are closely related to immune dysfunction in children with severe pneumonia, and once their IgA expression is abnormal, their respiratory mucosal barrier function is directly affected (29, 30) In response, clinical attention should be paid to the monitoring of IgA levels in children with severe pneumonia, with timely and effective measures been taken for intervention to improve airway clearance in children with severe pneumonia.

However, this study has certain limitations. For instance, as a single-center study with a small sample size, the limited number of participants may introduce selection bias, potentially affecting the representativeness and reliability of the findings. Additionally, the classification of ACD types in this study may differ from criteria used in other research, leading to limited comparability across studies. Therefore, future clinical efforts should focus on conducting multicenter, large-sample prospective studies to comprehensively understand the epidemiological characteristics, risk factors and prognosis of ACD in children with severe pneumonia. It is also essential to establish a unified and quantifiable classification standard for ACD types in this population to enhance the comparability and scientific rigor of research outcomes.

In summary, children with severe pneumonia are at risk of developing ACD, which may be influenced by factors such as mechanical ventilation, CRP, PCT, IL-6, and IgA levels. These five factors can be used to assess the risk of ACD in this population. If ACD is confirmed early through evaluation of these markers, clinical management should be promptly adjusted. Treatment strategies may include nebulized inhalation of mucolytic agents such as N-acetylcysteine to reduce sputum viscosity, supplementation with immunonutrients or intravenous immunoglobulin for children with low IgA levels, enhanced airway humidification, high-frequency oscillatory sputum expulsion, and daily assessment of weaning criteria to shorten mechanical ventilation duration, thereby reducing ventilator-induced lung injury.

## Data Availability

The original contributions presented in the study are included in the article/Supplementary Material, further inquiries can be directed to the corresponding author.
